# Within-Hospital Price Gaps Across National Insurers

**DOI:** 10.1001/jamanetworkopen.2024.51941

**Published:** 2024-12-23

**Authors:** Yang Wang, Jianhui Xu, Gerard Anderson

**Affiliations:** 1 Department of Health Policy and Management, Johns Hopkins Bloomberg School of Public Health, Baltimore, Maryland; 2 Department of International Health, Johns Hopkins Bloomberg School of Public Health, Baltimore, Maryland; 3 Johns Hopkins University School of Medicine, Baltimore, Maryland

## Abstract

**Importance:**

Commercial prices for hospital care are high and vary widely in the US. Employers and state policymakers are exploring reference-based pricing (RBP) to set their payment rates as multiples of Medicare prices; understanding the range of commercial price variation within a hospital is important for calculating the appropriate price targets that are effectively low to generate savings but also feasible and viable to local hospital markets.

**Objective:**

To examine within-hospital maximum-to-minimum commercial hospital price gaps negotiated by 5 national insurers and estimate plan savings if the minimum prices within each hospital are used as new payment level.

**Design, Setting, and Participants:**

This cross-sectional study used the insurer-disclosed Transparency in Coverage data as of March 2024. There were 40 382 commercial hospital facility prices extracted for 10 common services negotiated by CVS Health, Elevance Health, Blue Cross Blue Shield, Cigna, and United Healthcare, measured at the hospital-service-insurer level relative to the 2024 Medicare prices. For each hospital-service pair, the minimum, enrollment-weighted mean, and maximum prices were calculated, as well as the maximum-to-minimum price gaps. Plan savings were then estimated using the minimum within-hospital prices as the new payment levels.

**Exposure:**

Insurer price disclosure under federal Transparency in Coverage rule.

**Main Outcomes and Measures:**

Maximum-to-minimum commercial price gaps and estimated savings if using the minimum prices at hospital-service level.

**Results:**

Among 40 382 commercial hospital prices negotiated by 5 national insurers, the national means of minimum prices were 168% (95% CI, 167%-169%) of Medicare rates for inpatient services and 220% (95% CI, 215%-226%) of Medicare rates for outpatient services. National mean minimum-to-maximum price gaps were 86% (95% CI, 85%-87%) and 222% (95% CI, 215%-229%) of Medicare rates for inpatient and outpatient services, respectively. If using the minimum within-hospital prices, compared with current prices, payers could save 21% (95% CI, 20%-21%) for inpatient services and 29% (95% CI, 28%-30%) for outpatient services.

**Conclusions and Relevance:**

In this cross-sectional study, commercial prices across different national insurers varied substantially for the same hospital and service. These results suggest that employers and policymakers interested in RBP benchmarking may use the lowest prices among major insurers in their local hospital market as references to negotiate lower prices.

## Key Points

**Question:**

In Medicare reference-based pricing, how should price targets that are effectively low to generate savings but also feasible to local hospital markets be chosen?

**Findings:**

In this cross-sectional study of 40 382 commercial prices negotiated by 5 national insurers, the lowest mean within-hospital prices were 168% and 220% of Medicare rates for inpatient and outpatient services, respectively; and estimated 21% savings for inpatient services and 29% savings for outpatient services could be achieved by using the lowest within-hospital prices as new payment levels.

**Meaning:**

These results suggest that the lowest within-hospital prices negotiated by national insurers can be used as price targets to generate savings.

## Teaser

This cross-sectional study investigates within-hospital maximum-to-minimum price gaps negotiated by 5 national insurers and estimates plan savings if each hospital used its minimum prices as the new payment level.

## Introduction

Commercial negotiated prices for hospital care are high and vary widely in the US.^1,2^ Over the past 2 decades, they have increased more than 200%, a magnitude far exceeding other consumer goods and services.^3^ As of 2022, mean commercial prices have reached 254% of Medicare rates for the same hospital and service.^4^ To address this affordability crisis among commercial patients, a growing number of self-insured employers, purchasing coalitions, and state public option plans are exploring Medicare reference-based pricing (RBP), which set their plans’ payment rates at a multiple (eg, 200%) of Medicare rates.^5,6,7^ A key success factor of this price benchmarking effort is to find out the appropriate commercial price targets that are effectively low to generate sizable savings but also viable and feasible to the local hospital markets (eg, not too low to undermine hospitals’ financial viability, operation, or patient access).^8^ Therefore, understanding the full range of commercial price variation, especially across different insurers for the same service delivered in the same hospital, benchmarked against Medicare rates, can help inform this price target calculation. Yet, little empirical work has been done in this regard. One major barrier is the nontransparent commercial price negotiation process, which prevents patients, employers, and policymakers from accessing necessary pricing information to effectively compare, negotiate, or regulate their payments.^4^

Effective in July 2022, the federal Transparency in Coverage (TiC) Final Rule required all commercial insurers to publicly disclose their negotiated prices for specific procedures and contracting health care organizations and clinicians.^9^ Leveraging this up-to-date, nationally comprehensive, and granular price data, we examine within-hospital commercial price variation across 5 national insurers for 10 common hospital services. We calculate the maximum-to-minimum price gaps at each hospital and service, relative to Medicare rates, and then estimate the corresponding savings if the minimum commercial prices at each hospital are used as the target payment level for RBP benchmark. Our study aims to shed light on the dynamics of within-hospital commercial pricing and support employers and policymakers interested in RBP with recommended price targets and estimated savings.

## Methods

This cross-sectional study followed the Strengthening the Reporting of Observational Studies in Epidemiology (STROBE) reporting guideline. Institutional review board approval and informed consent were not sought because this research does not involve human participants.

### Data and Sample

Our primary data source was the insurer-disclosed price transparency data as of March 2024.^9^ Specifically, we accessed the data from Turquoise Health, a third-party data platform that collected pricing data disclosed by individual insurers in compliance with the TiC rule, and compiled them into a standardized price database with corresponding insurer, procedure, and health care organization or clinician information.^10^ This TiC price transparency data has been cross validated by multiple studies and used in recent research on commercial pricing for hospital care.^11,12,13,14^ Specifically, we extracted commercial hospital facility prices negotiated by 5 national insurers, including CVS Health, Elevance Health, Blue Cross Blue Shield (BCBS), Cigna, and UnitedHealthcare, accounting for 78% of the total commercial market enrollment in 2022.^15^ Following prior research on hospital pricing, we focused on 10 common hospital services, including percutaneous cardiovascular procedures (PTCA), spinal fusion, hip and knee replacement, cellulitis, cesarean section, normal delivery, septicemia, and psychosis in the inpatient setting, and esophagogastroduodenoscopy (EGD) and colonoscopy that were mostly delivered in the outpatient setting (more details in eTable 1 in Supplement 1).^1,11,16^ To create a standardized price measure, we excluded insurer-disclosed prices measured as percentages or on per diem basis, and then constructed our price measures at hospital-services-insurer level, using the median values if multiple prices were reported (eg, across different plans within an insurer). Given the lack of utilization measure and potential duplicated data points, this method enabled us to consistently measure and compare prices across different insurers, services, and hospitals.^14,17^

We then scaled all commercial prices as percentages relative to Medicare rates for the same hospital and service, using the 2024 Inpatient and Outpatient Prospective Payment System (IPPS/OPPS) parameters published by the Centers for Medicare & Medicaid Services (CMS).^18^ We limited our hospital samples to general acute care hospitals identified in the American Hospital Association (AHA)’s annual survey of 2022, after excluding critical access hospitals and Maryland hospitals because they were not paid under the IPPS/OPPS mechanism.^19^ Since this study examined within-hospital price variation, we kept hospitals with prices disclosed by at least 2 national insurers. Although the 5 national insurers combined accounted for approximately 80% of total commercial market enrollment, some of them may have smaller commercial market penetration in some local areas. Therefore, to ensure our price samples were all representative of sizable commercial market enrollment, we only included prices when the disclosing insurer had at least 5% commercial market share in the local hospital referral region (HRR), identified using the 2023 Interstudy Insurance Enrollment data.^20^ As a sensitivity analysis, we reran our analysis by increasing the HRR level enrollment share threshold to 10%. For each procedure, top and bottom 1% commercial prices (relative to Medicare rates) were further excluded as potential data errors.^14^

### Statistical Analysis

To document the extent of commercial price variation within a hospital, we first identified the minimum, weighted mean (by each insurer’s HRR-level commercial enrollment share), and maximum prices for each hospital-service pair, relative to Medicare rates. We calculated the national means for these 3 price measures for each service. We further explored geographic heterogeneity using heat maps to show the varying levels of minimum prices averaged at the HRR level across the nation.

We then calculated the maximum-to-minimum price gaps for each hospital-service pair, relative to Medicare rates. We examined the mean gaps for each service at the national level, as well as HRR level using heat maps. Next, we estimated potential plan savings if the minimum within-hospital prices were used as the new RBP benchmark for each service. Specifically, we calculated the relative percentage savings across all insurers at each hospital as the relative price difference between the new RBP benchmark and the current enrollment-weighted mean price. We then summarized the mean savings both at the national and HRR level.

Presto SQL language and DBeaver software were used to access data from Turquoise Health’s platform. STATA version 17 (StataCorp) was used for data analysis from May to July 2024. Two-sided *t* tests were used to calculate statistical significance (*P* < .05).

## Results

Our study examined 40 382 prices measured at the hospital-services-insurer level, corresponding to 14 757 hospital-service level maximum-to-minimum price gaps. Our sample represented 1970 general acute care noncritical access hospitals located in 44 states and 257 HRRs, accounting for 67% of all such hospitals in the US. Among them, 614 (31%) had the minimum prices consistently negotiated by the same insurer across all 10 services. While no single national insurer negotiated lowest prices in all hospital-service pairs, BCBS and Elevance Health negotiated the lowest prices more frequently than when they paid the highest prices (26% vs 16%, 9% vs 7%, respectively) (eFigure 1 in Supplement 1). In contrast, UnitedHealthcare, Cigna, and CVS Health paid the lowest prices less frequently than paying the highest prices (33% vs 37%, 14% vs 17%, 17% vs 23%, respectively). Meanwhile, BCBS and Elevance Health accounted for 31% and 15% of commercial market share, followed by UnitedHealthcare (14%), Cigna (10%), and CVS Health (9%).

**Figure 1.**
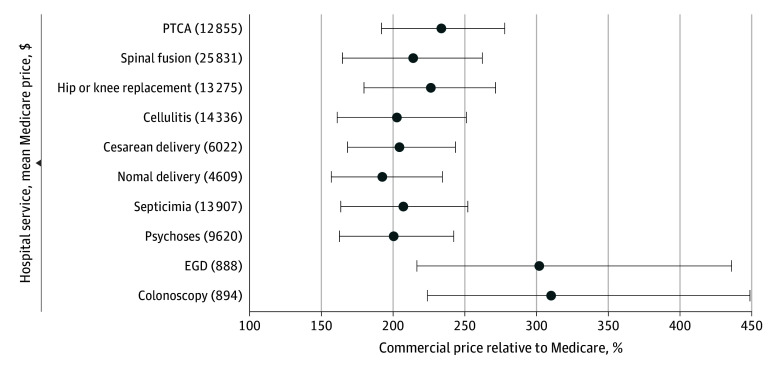
National Mean of the Within-Hospital Minimum, Enrollment-Weighted Mean, and Maximum Prices Relative to Medicare Dots represent the enrollment-weighted mean, and whiskers represent the national mean of minimum and maximum prices. EGD indicates esophagogastroduodenoscopy; PTCA, percutaneous cardiovascular procedures.

The national means of the within-hospital minimum, weighted mean, and maximum prices for the 8 inpatient services were 168% (95% CI, 167%-169%), 210% (95% CI, 209%-211%), and 254% (95% CI, 253%-256%) of Medicare rates, respectively. Specifically, normal delivery had the lowest rates, which were 157% (95% CI, 154%-160%), 192% (95% CI, 189%-195%), and 234% (95% CI, 231%–238%) of Medicare rates for minimum, weighted mean, and maximum prices, respectively (Figure 1; eTable 2 in Supplement 1). On the other hand, PTCA had the highest minimum prices (192% [95% CI, 187%-196%]), weighted mean prices (234% [95% CI, 229%-238%]), and maximum prices (278% [95% CI, 272%-283%]).

In contrast, the 2 outpatient services had systematically higher minimum, weighted mean, and maximum prices, corresponding to 220% (95% CI, 215%-226%), 306% (95% CI, 300%-312%), and 442% (95% CI, 434%-451%) of Medicare rates, respectively. Specifically, colonoscopy had higher prices than EGD: 224% (95% CI, 216%-232%) vs 217% (95% CI, 209%-224%) for minimum prices; 310% (95% CI, 301%-319%) vs 302% (95% CI, 293%-311%) for weighted mean prices, and 449% (95% CI, 436%-461%) vs 436% (95% CI, 424%-448%) for maximum prices. Although the mean prices for each service were uniquely distinct in dollar amounts (eTable 1 in Supplement 1), the mean minimum prices, relative to Medicare, were consistent among the 8 inpatient services, and between the 2 outpatient procedures.

**Figure 2.**
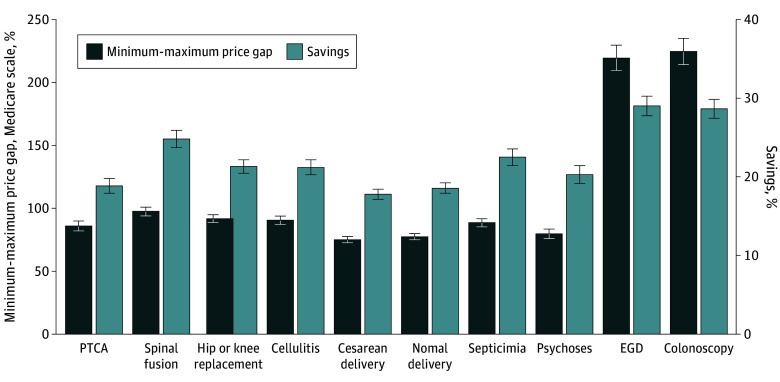
National Mean of the Maximum-to-Minimum Price Gap and Estimated Savings EGD indicates esophagogastroduodenoscopy; PTCA, percutaneous cardiovascular procedures. Whiskers represent 95% CIs.

Figure 2 summarizes the national means of the maximum-to-minimum price gaps for each service on the left y-axis. The mean price gaps were 86% (95% CI, 85%-87%) of Medicare rates for the 8 inpatient services combined, where spinal fusion had the largest gaps (97% [95% CI, 94%-101%] of Medicare rate) and cesarean section had the smallest gaps (75% [95% CI, 73%-78%] of Medicare rate). The mean price gaps were much larger for outpatient procedures (222% [95% CI, 215%-229%] of Medicare rate), where colonoscopy had slightly wider gaps than EGD (225% [95% CI, 214%-235%] vs 220% [95% CI, 209%-230%]). Figure 2 (right y-axis) and eFigure 2 in Supplement 1 also show the estimated percentage savings when the minimum within-hospital prices were used as the new payment levels for each service. Compared with current prices, we estimated a mean of 21% (95% CI, 20%-21%) savings for inpatient services, with the largest saving from spinal fusion (25% [95% CI, 24%-26%]) and smallest saving from cesarean delivery (18% [95% CI, 17%-18%]). We also estimated a mean of 29% (95% CI, 28%-30%) for outpatient services, with very similar magnitude of savings for colonoscopy and EGD.

**Figure 3.**
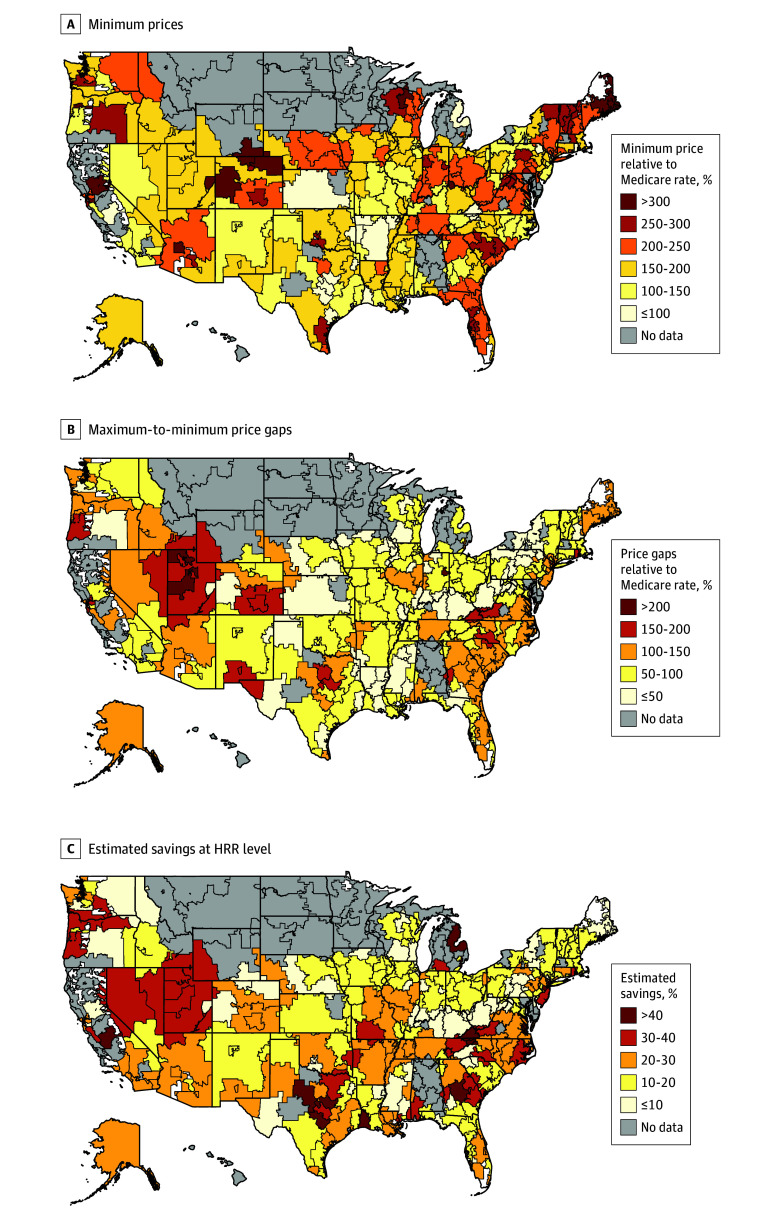
Pricing and Saving Dynamics Across 249 Hospital Referral Regions (HRRs) for Hip and Knee Replacement Services Minimum prices (A), maximum-to-minimum price gaps (B), and estimated savings (C) at HRR level for hip and knee replacement. Minimum prices and price gaps are measured relative to Medicare rates. Estimated savings are measured in percentages. All results are averaged across hospitals within each HRR.

The heat maps in Figure 3 illustrate the pricing and saving dynamics across 249 HRRs for hip and knee replacement, a common inpatient service among both commercial patients and Medicare beneficiaries. Figure 3A shows substantial variation in minimum prices across HRRs, ranging from slightly less than 100% (19 HRRs) to more than 300% (10 HRRs) of Medicare rates. The price gaps also varied widely, ranging from less than 50% (53 HRRs) to more than 200% (3 HRRs) of Medicare rates (Figure 3B). Moreover, HRRs with wider price gaps would experience larger savings, indicated by a correlation of 0.67. Specifically, 16 HRRs would experience more than 40% savings, the largest saving category in Figure 3C, after using the minimum within-hospital prices as the new payment level. These findings were robust across other hospital services (eFigure 3 and eFigure 4 in Supplement 1). Sensitivity analyses excluding national insurers with less than 10% of enrollment in each HRR had similar results (eTable 3 in Supplement 1).

## Discussion

This study found substantial payment gaps between the maximum and minimum prices within a hospital negotiated by different national insurers, equivalent to nearly 100% of Medicare rates for inpatient services and more than 200% for outpatient procedures. Given these wide payment gaps, up to 30% in savings could be achieved if a plan uses the lowest within-hospital negotiated prices as the new payment level. Our findings contribute to a growing body of literature examining variation in commercial hospital prices,^1,4,21^ but using a novel, comprehensive, and granularly measured data source not previously available. Furthermore, our findings support employers, commercial plans, and policymakers exploring options to lower commercial hospital prices by informing them of the pricing dynamics in their local hospital markets, especially the lowest price points.

Recently, state employee plans from Oregon, North Carolina, and Montana, as well as state public option plans issued in Washington, Colorado, and Nevada have successfully implemented Medicare reference-based pricing to generate savings for their plans and enrollees by setting their prices ranging from 155% to 250% of Medicare rates.^5,6,7,22^ State employers and plans may use the lowest payment levels estimated from this study as reference points to negotiate lower prices and generate savings in their local areas, as this approach has the following strengths: first, instead of implementing a new payment level with unknown impacts, this approach uses current prices already accepted by hospital markets and widely applied to commercial patients enrolled under 1 of the 5 national insurers; second, by focusing on reducing within-hospital price gaps, this approach does not change insurance network sizes or steer patients to certain health care organizations than others such as the reference pricing model implemented by the California Public Employees Retirement System (CalPERS),^23,24^ which might incur potential disruptions on patient access; and third, our proposed payment benchmarks are easy to calculate. While the lowest market prices within a hospital vary widely across procedures, our proposed price benchmarks are essentially measured (and expressed) as multiples of Medicare rates, which are quite consistent across different services within inpatient or outpatient settings. Therefore, a plan may set 2 payment levels, one for inpatient and another for outpatient care, instead of setting unique dollar-based payment levels for each individual hospital service.

Admittedly, knowing the lowest market prices does not necessarily guarantee that such prices can be obtained by all other plans through price match, as negotiated prices are often influenced by the relative bargaining power between payers and hospitals in the local market, as well as by government regulations on pricing.^1,8,25,26^ In addition, potential impacts from reduced prices on patients’ network access and treatment outcomes remain unknown and warrant future research.

Nevertheless, our findings equip employers and purchasers with the necessary information to explore strategies to lower their prices for hospital care, even though obtaining the lowest prices might not always be feasible. Unlike most other consumer markets, commercial prices for hospital care have long been opaque, leaving patients and employers uninformed and disadvantaged when paying for hospital services.^4^ Moreover, nearly two-thirds of the employer-sponsored plans are self-insured, where the contracting insurers do not bear risk, and, therefore, may have diminished incentive to negotiate lower prices for their patients and employers,^25,27^ Therefore, our empirical findings have broad relevance to all employer plans, regardless of their negotiating leverage. Specifically, our results encourage employers to actively assess their plans’ current payment rates, compare them with the lowest price points in their local market, and explore options on lowering their payments. Such options may include shopping across different contracting insurers, engaging in direct contracting with health care organizations, or strengthening their collective bargaining power by forming purchasing coalitions.^28,29^ Meanwhile, continuing policy efforts on further strengthening the access, quality, and usability of the price transparency data are necessary to support this price comparison and negotiation strategy in commercial hospital markets.^30^

### Limitations

Our study has limitations. First, the TiC price transparency data are contingent on insurer’s disclosure and may be subject to measurement inaccuracies. Our price measure, which is aggregated at the hospital-services-insurer level, masks the more granular, plan-specific price heterogeneity. Second, by focusing on hospital facility prices for 10 common services negotiated by 5 national insurers, our findings might not be fully generalizable to other services, health care organization settings, or insurers. Third, by measuring commercial prices relative to Medicare rates, our results can be influenced by Medicare’s payment level, which varies across procedures (eg, higher payments for newborn delivery services due to more complications among Medicare patients with disabilities). Fourth, our estimated savings are under static assumptions and do not account for variation in insurance network, care utilization, outcome, or patient characteristics due to data limitation. Whether and how these factors influence the commercial price gaps are important questions that warrant future research.

## Conclusions

Commercial prices within a hospital vary considerably when negotiated by different national insurers. Employers and policymakers interested in lowering their plans’ payment rates should compare their current prices relative to the lowest prices negotiated among national insurers in their local hospital market. Conditional on their bargaining leverage, they may use the lowest price points as references to obtain lower negotiated prices and generate savings for patients.

## Notes

Supplement 1. **eFigure 1.** Distribution of National Insurers Negotiating the Minimum Prices and the Maximum Prices **eFigure 2.** National Average of the Estimated Savings for Each Service, Measured as Percentage of Medicare Rates **eFigure 3.** Minimum Prices, Maximum-to-Minimum Price Gaps, and Estimated Savings at Hospital Referral Region (HRR) Level for Normal Delivery **eFigure 4.** Minimum Prices, Maximum-to-Minimum Price Gaps, and Estimated Savings at Hospital Referral Region (HRR) Level for Colonoscopy **eTable 1.** Description of Procedures, and Their Corresponding National Average Prices **eTable 2.** National Average and 95% Confidence Interval of the within-Hospital Minimum, Enrollment-weighted Average, and Maximum Prices, Relative to Medicare **eTable 3.** National Average of the within-Hospital Minimum Prices, Enrollment-weighted Average Prices, Maximum Prices, Minimum-to-Maximum Price Gaps, and Estimated Savings after Excluding National Insurers with Less than 10% Market Share at Hospital Referral Region (HRR) Level

Supplement 2.
Data Sharing Statement
