# Supplementary material for: Within-Hospital Price Gaps Across National Insurers
Source: JAMA Netw Open. 2024 Dec 23;7(12):e2451941. doi: 10.1001/jamanetworkopen.2024.51941 (PMC11667364; doi:10.1001/jamanetworkopen.2024.51941)
Supplement: Supplement 1. — eFigure 1. Distribution of National Insurers Negotiating the Minimum Prices and the Maximum Prices eFigure 2. National Average of the Estimated Savings for Each Service, Measured as Percentage of Medicare Rates eFigure 3. Minimum Prices, Maximum-to-Minimum Price Gaps, and Estimated Savings at Hospital Referral Region (HRR) Level for Normal Delivery eFigure 4. Minimum Prices, Maximum-to-Minimum Price Gaps, and Estimated Savings at Hospital Referral Region (HRR) Level for Colonoscopy eTable 1. Description of Procedures, and Their Corresponding National Average Prices eTable 2. National Average and 95% Confidence Interval of the within-Hospital Minimum, Enrollment-weighted Average, and Maximum Prices, Relative to Medicare eTable 3. National Average of the within-Hospital Minimum Prices, Enrollment-weighted Average Prices, Maximum Prices, Minimum-to-Maximum Price Gaps, and Estimated Savings after Excluding National Insurers with Less than 10% Market Share at Hospital Referral Region (HRR) Level [file jamanetwopen-e2451941-s001.pdf]

Supplemental Online Content

Wang Y, Xu J, Anderson G. Within-hospital price gaps across national insurers. *JAMA Netw Open.* 2024;7(12):e2451941. doi:10.1001/jamanetworkopen.2024.51941

eFigure 1. Distribution of National Insurers Negotiating the Minimum Prices and the Maximum Prices

eFigure 2. National Average of the Estimated Savings for Each Service, Measured as Percentage of Medicare Rates

eFigure 3. Minimum Prices, Maximum-to-Minimum Price Gaps, and Estimated Savings at Hospital Referral Region (HRR) Level for Normal Delivery

eFigure 4. Minimum Prices, Maximum-to-Minimum Price Gaps, and Estimated Savings at Hospital Referral Region (HRR) Level for Colonoscopy

eTable 1. Description of Procedures, and Their Corresponding National Average Prices

eTable 2. National Average and 95% Confidence Interval of the within-Hospital Minimum, Enrollment-weighted Average, and Maximum Prices, Relative to Medicare

eTable 3. National Average of the within-Hospital Minimum Prices, Enrollment-weighted Average Prices, Maximum Prices, Minimum-to-Maximum Price Gaps, and Estimated Savings after Excluding National Insurers with Less than 10% Market Share at Hospital Referral Region (HRR) Level

This supplementary material has been provided by the authors to give readers additional information about their work.

eFigure 1. Distribution of National Insurers Negotiating the Minimum Prices and the Maximum Prices

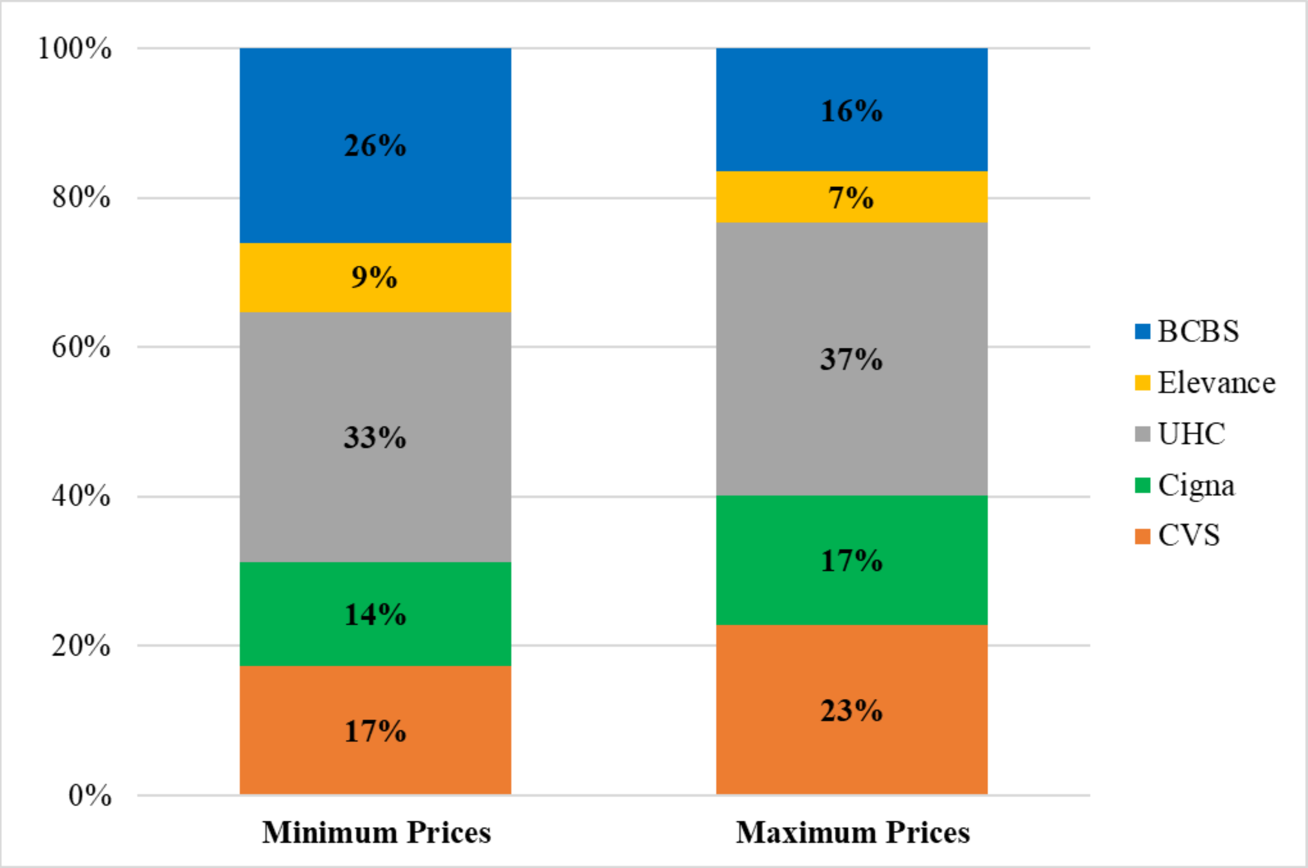

Note: Under Chi-sq test, the difference between these two distributions is statistically significant ($P < 0.001$). CVS Health, Elevance Health, BCBS, Cigna, and UHC accounted for 9%, 15%, 31%, 10%, 14% of commercial market share among the 257 hospital referral regions included in this study, respectively. UHC United Healthcare.

© 2024 Wang Y et al. *JAMA Network Open*.

eFigure 2. National Average of the Estimated Savings for Each Service, Measured as Percentage of Medicare Rates

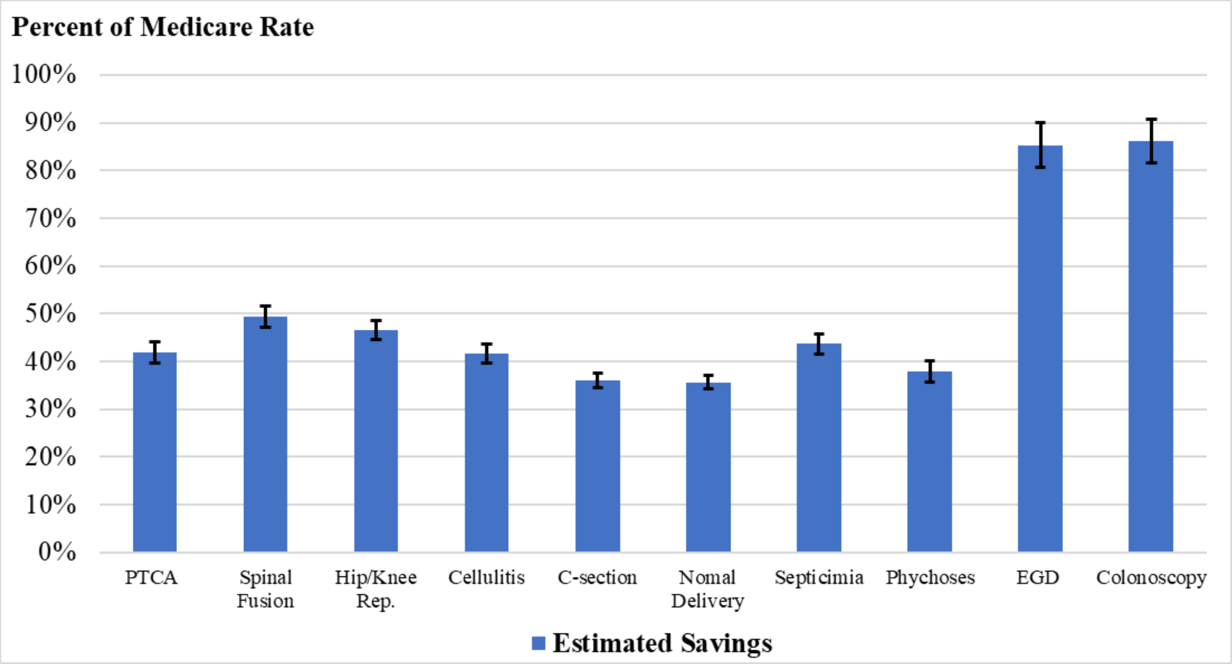

Note: PTCA, percutaneous cardiovascular procedures; Hip/Knee Rep., hip and knee replacement; C-section, cesarean section; EGD, esophagogastroduodenoscopy. 95% confidence intervals are marked as error bars.

© 2024 Wang Y et al. *JAMA Network Open*.

eFigure 3. Minimum Prices, Maximum-to-Minimum Price Gaps, and Estimated Savings at Hospital Referral Region (HRR) Level for Normal Delivery

Panel A Minimum Prices

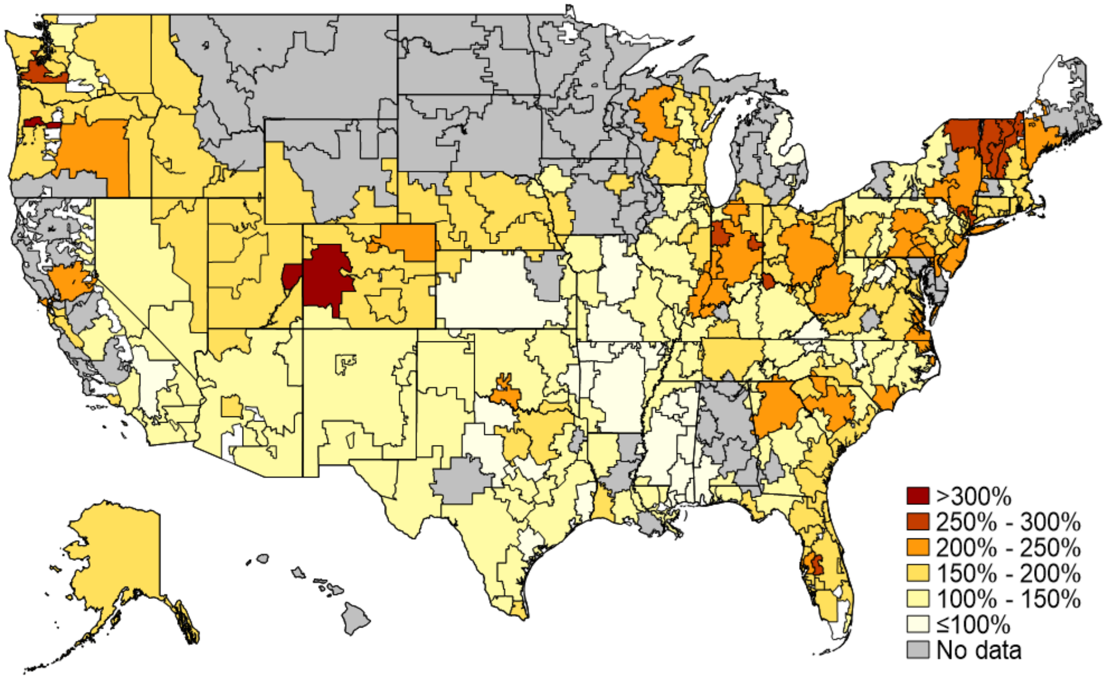

Panel B Maximum-to-Minimum Price Gaps

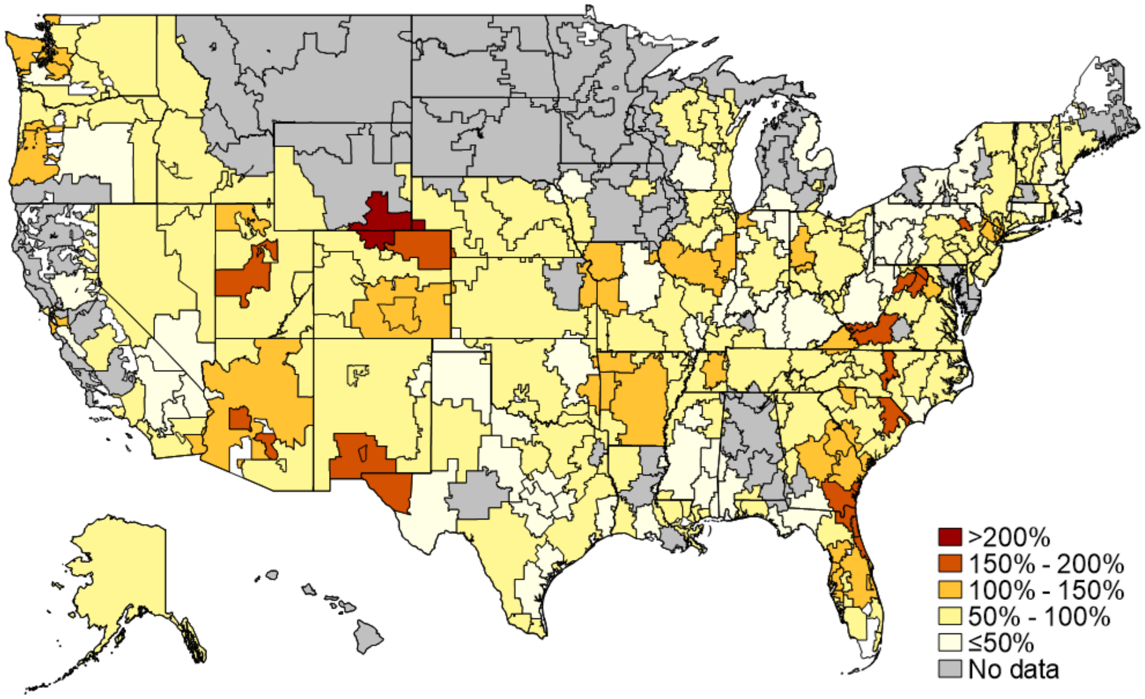

© 2024 Wang Y et al. *JAMA Network Open*.

Panel C Estimated Savings

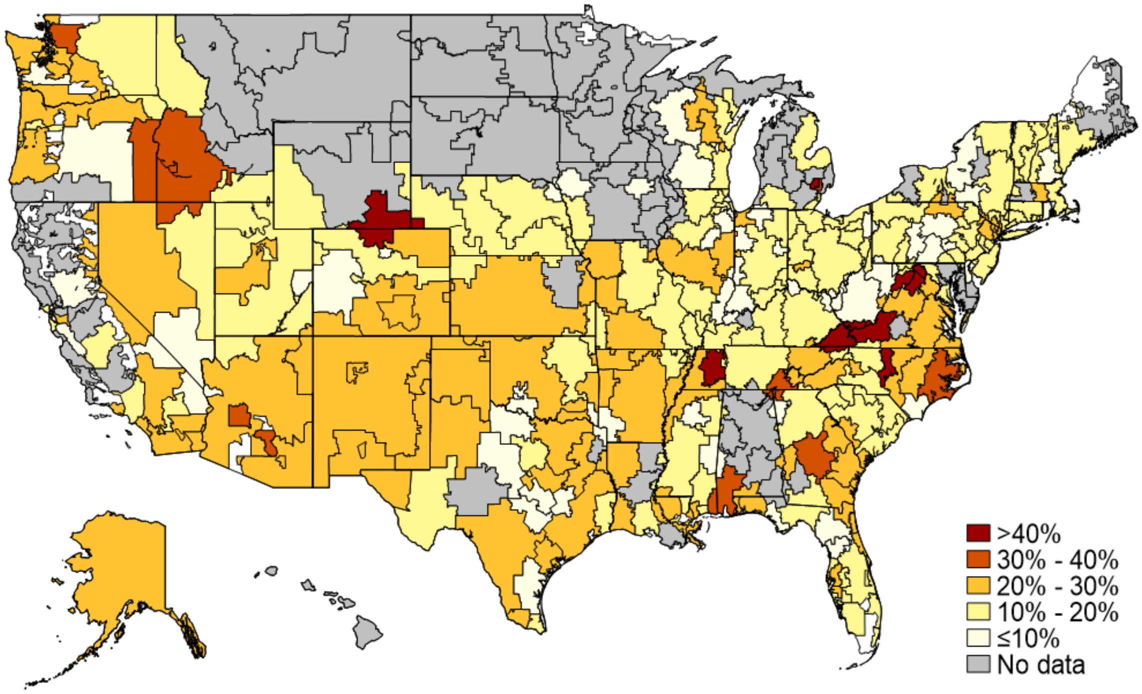

Note: Minimum prices and price gaps are measured relative to Medicare rates. Estimated savings are measured in percentages. All results are averaged across hospitals within each HRR.

© 2024 Wang Y et al. *JAMA Network Open*.

eFigure 4. Minimum Prices, Maximum-to-Minimum Price Gaps, and Estimated Savings at Hospital Referral Region (HRR) Level for Colonoscopy

Panel A Minimum Prices

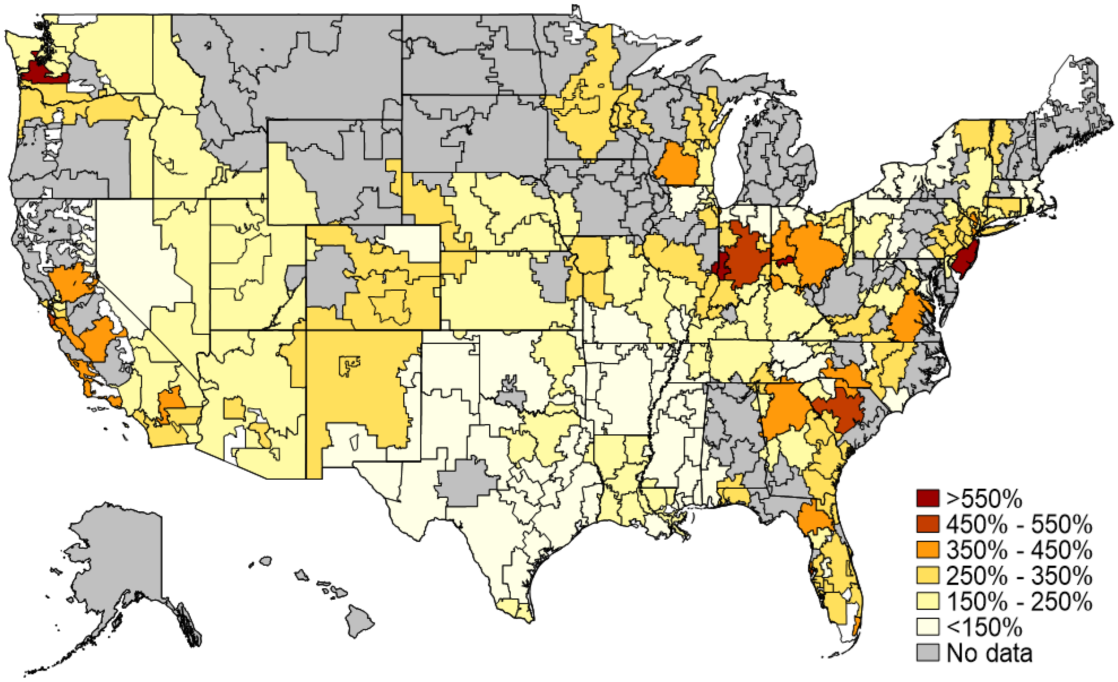

Panel B Maximum-to-Minimum Price Gaps

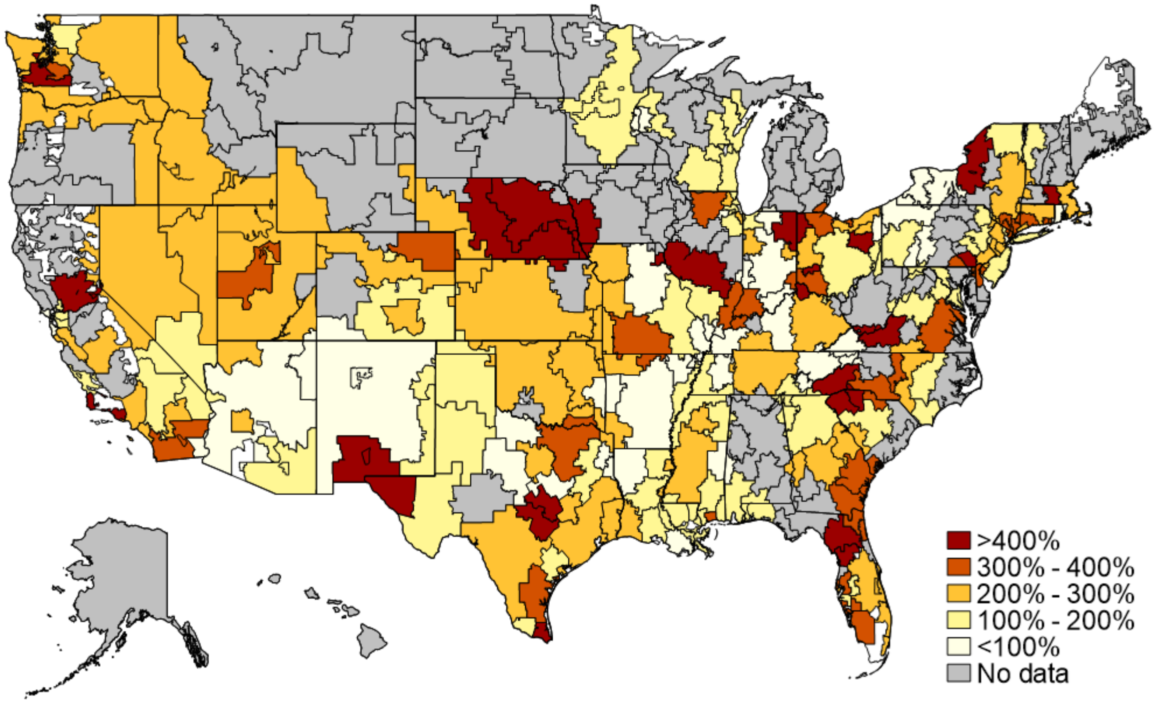

© 2024 Wang Y et al. *JAMA Network Open*.

Panel C Estimated Savings

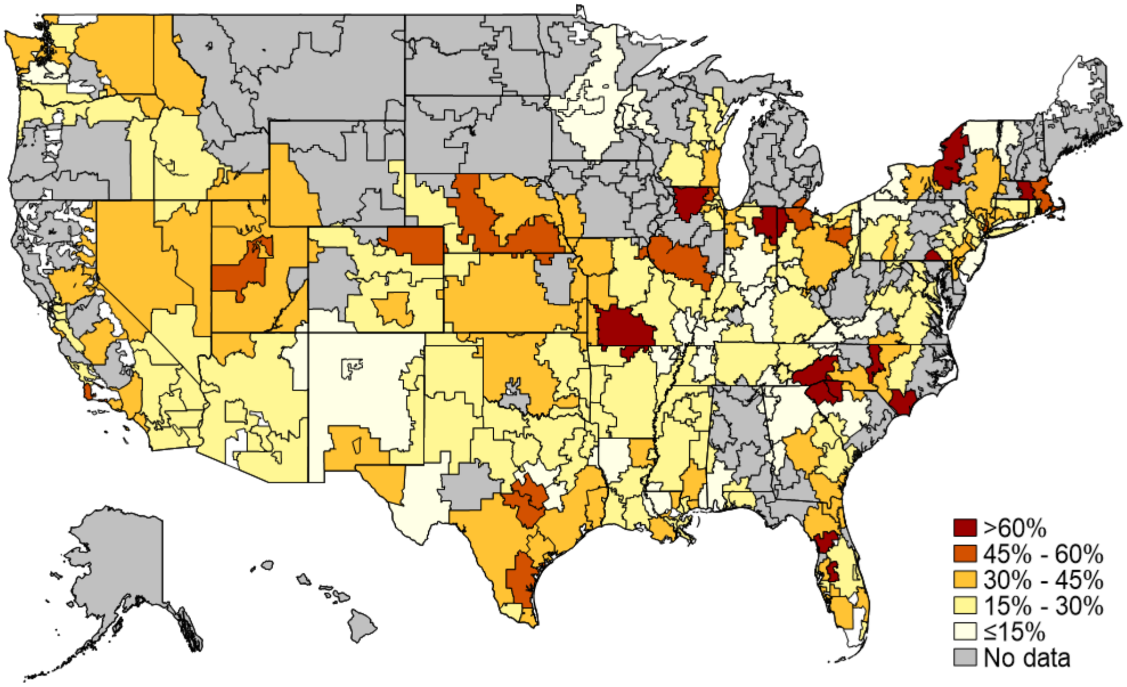

Note: Minimum prices and price gaps are measured relative to Medicare rates. Estimated savings are measured in percentages. All results are averaged across hospitals within each HRR.

© 2024 Wang Y et al. *JAMA Network Open*.

eTable 1. Description of Procedures, and Their Corresponding National Average Prices

Procedure Name	Procedure Code	Average Commercial Price	Average Medicare Price	Average Ratio
Percutaneous Cardiovascular Procedures (PTCA)	DRG 322	$30,752	$12,855	2.37
Spinal Fusion	DRG 460	$56,441	$25,831	2.18
Hip and Knee Replacement	DRG 470	$30,695	$13,275	2.31
Cellulitis	DRG 575	$30,492	$14,336	2.12
Cesarean Section	DRG 788	$12,782	$6,022	2.11
Normal Delivery	DRG 807	$9,288	$4,609	2.00
Septicemia	DRG 871	$29,858	$13,907	2.14
Psychosis	DRG 885	$19,814	$9,620	2.05
Esophagogastroduodenoscopy (EGD)	CPT 43235	$2,926	$888	3.26
Colonoscopy	CPT 45378	$3,039	$894	3.36

Note: To standardize price measure, procedures are identified using the corresponding base DRG codes or base CPT codes (e.g. no complication or comorbidity [CC], major complication or comorbidity [MCC], or add-on services). Price samples with billing code modifiers are also excluded. DRG diagnostic-related group, CPT current procedural terminology.

© 2024 Wang Y et al. *JAMA Network Open*.

eTable 2. National Average and 95% Confidence Interval of the within-Hospital Minimum, Enrollment-weighted Average, and Maximum Prices, Relative to Medicare

Procedure Name	Minimum	Weighted Average	Maximum
Percutaneous Cardiovascular Procedures (PTCA)	192% (187%, 196%)	234% (229%, 238%)	278% (272%, 283%)
Spinal Fusion	165% (161%, 169%)	214% (210%, 218%)	262% (258%, 266%)
Hip and Knee Replacement	180% (176%, 183%)	226% (223%, 230%)	271% (267%, 276%)
Cellulitis	161% (157%, 165%)	203% (199%, 206%)	252% (247%, 256%)
Cesarean Section	168% (165%, 171%)	204% (201%, 207%)	243% (240%, 247%)
Normal Delivery	157% (154%, 160%)	192% (189%, 195%)	234% (231%, 238%)
Septicemia	163% (160%, 167%)	207% (204%, 211%)	252% (248%, 256%)
Psychosis	162% (158%, 167%)	200% (196%, 205%)	242% (238%, 247%)
Esophagogastroduodenoscopy (EGD)	217% (209%, 224%)	302% (293%, 311%)	436% (424%, 448%)
Colonoscopy	224% (216%, 232%)	310% (301%, 319%)	449% (436%, 461%)

Note: 95% confidence interval in parenthesis. All price measures are relative to Medicare rates in percentage.

© 2024 Wang Y et al. *JAMA Network Open*.

eTable 3. National Average of the within-Hospital Minimum Prices, Enrollment-weighted Average Prices, Maximum Prices, Minimum-to-Maximum Price Gaps, and Estimated Savings after Excluding National Insurers with Less than 10% Market Share at Hospital Referral Region (HRR) Level

Procedure Name	Procedure Code	Minimum Price	Weighted Average Price	Maximum Price	Min-Max Price Gap	Estimated Savings
Percutaneous Cardiovascular Procedures (PTCA)	DRG 322	192%	231%	275%	82%	18%
Spinal Fusion	DRG 460	176%	219%	262%	86%	22%
Hip and Knee Replacement	DRG 470	190%	232%	272%	81%	19%
Cellulitis	DRG 575	169%	208%	252%	83%	20%
Cesarean Section	DRG 788	174%	207%	240%	66%	16%
Normal Delivery	DRG 807	161%	193%	229%	68%	17%
Septicemia	DRG 871	171%	211%	251%	80%	21%
Psychosis	DRG 885	164%	201%	242%	78%	20%
Esophagogastroduodenoscopy (EGD)	CPT 43235	215%	289%	402%	188%	26%
Colonoscopy	CPT 45378	222%	296%	407%	186%	26%

Note: All prices are measured relative to Medicare rates. Estimated savings are measured in percentages.

© 2024 Wang Y et al. *JAMA Network Open.*
