# Supplementary material for: Within-Hospital Price Gaps Across National Insurers
Source: JAMA Netw Open. 2024 Dec 23;7(12):e2451941. doi: 10.1001/jamanetworkopen.2024.51941 (PMC11667364; doi:10.1001/jamanetworkopen.2024.51941)
Supplement: Supplement 2. — Data Sharing Statement [file jamanetwopen-e2451941-s002.pdf]

Data Sharing Statement

Wang. Within-Hospital Price Gaps Across National Insurers. *JAMA Netw Open*. Published December 23, 2024. doi:10.1001/jamanetworkopen.2024.51941

Data
Data available: Yes
Data types: Data (not involving human participants)
How to access data: Data request through email: ywang406@jhu.edu
When available: With publication

Supporting Documents
Document types: None

Additional Information
Who can access the data: anyone requesting the data
Types of analyses: For purposes related to promoting healthcare price transparency, encouraging price competition and negotiation, supporting policymaking that aims to lower commercial healthcare prices
Mechanisms of data availability: No specific mechanism
Any additional restrictions: NA
